# Benefit and risk of early intravenous heparin after thrombolysis in patients with acute ischemic stroke

**DOI:** 10.1002/brb3.1776

**Published:** 2020-09-05

**Authors:** Xu Tong, Yibin Cao, Wenzhi Wang, David Wang, Yongjun Wang, Yilong Wang, the Thrombolysis Implementation, Monitor of Acute Ischemic Stroke in China (TIMS‐China) Investigators

**Affiliations:** ^1^ Department of Interventional Neuroradiology Beijing Tiantan Hospital Capital Medical University Beijing China; ^2^ Beijing Neurosurgical Institute Capital Medical University Beijing China; ^3^ Department of Neurology Tangshan Gongren Hospital Hebei Medical University Tangshan China; ^4^ Illinois Neurological Institute Stroke Network Sisters of the Third Order of St Francis Healthcare System University of Illinois College of Medicine Peoria IL USA; ^5^ Department of Neurology Beijing Tiantan Hospital Capital Medical University Beijing China; ^6^ China National Clinical Research Center for Neurological Diseases Beijing Tiantan Hospital Capital Medical University Beijing China

**Keywords:** anticoagulation, heparin, ischemic stroke, thrombolysis, tissue plasminogen activator

## Abstract

**Background and purpose:**

We performed a retrospective analysis of the “Thrombolysis Implementation and Monitor of Acute Ischemic Stroke in China (TIMS‐China)” registry to explore the benefit and risk of intravenous thrombolysis (IVT) followed by intravenous heparin (IVH) in acute ischemic stroke (AIS) patients.

**Methods:**

In the TIMS‐China database, the patients who received IVH immediately after IVT (Early IVH group) and those who initiated antithrombotic therapy (ATT) until 24 hr after IVT (Standard ATT group) were screened for this comparison. Propensity score (PS) matching was performed between both groups. The logistic regression analysis was performed in the matched population to compare all the efficacy and safety outcomes.

**Results:**

Of 1,437 patients in this study, 119 received early IVH and 1,318 cases initiated standard ATT. After PS matching (1:2), 117 pairs were identified. The early IVH group had higher proportions of neurological improvement at 24 hr (OR = 2.24, 95% CI = 1.42–3.53) and 7 days (OR = 1.92, 95% CI = 1.22–3.03), better chance of excellent recovery (OR = 1.69, 95% CI = 1.07–2.67) and functional independence (OR = 1.77, 95% CI = 1.13–2.78) at 90 days, and a lower 90‐day mortality (OR = 0.44, 95% CI = 0.21–0.92) than standard ATT group. Additionally, early IVH did not increase the risk of symptomatic intracranial hemorrhage (OR = 0.92, 95% CI = 0.34–2.48).

**Conclusions:**

IVH immediately after thrombolysis seems to be safe and potentially more effective as compared with standard ATT delay of 24 hr for a subset of AIS patients.

## Graphical abstract

We aimed to explore the risk and benefit of bridging intravenous heparin (IVH) after intravenous thrombolysis (IVT) in patients with acute ischemic stroke (AIS). Therefore, we conducted this propensity score matched‐control study by using the data from the Thrombolysis Implementation and Monitor of Acute Ischemic Stroke in China (TIMS‐China). Our results suggested that in this cohort of AIS patients, such combination therapy with IVT followed by IVH might improve their functional outcomes and not increase their risk of hemorrhagic transformation.

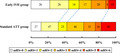

## 1 INTRODUCTION

Intravenous tissue plasminogen activator (tPA) is still the first‐line therapy for patients with acute ischemic stroke (AIS) within 4.5 hr after symptom onset, regardless of the availability of endovascular treatment. (Powers, Rabinstein, & Ackerson, 2018) The current guideline recommends withholding antithrombotic therapy (ATT) for 24 hr after intravenous tPA (IVT). (Powers et al., 2018) The tPA has a very short half‐life (about 5 min), and during the first 24‐hr post‐IVT treatment, this short half‐life may have contributed to the facts of low rate of recanalization,(Muchada et al., 2014; Rha & Saver, 2007; Yeo et al., 2013) early reocclusion,(Alexandrov & Grotta, 2002; Rubiera et al., 2005) IVT‐activated hypercoagulation,(Fassbender et al., 1999), and downstream thromboses in the microvasculature from the broken‐up thrombi after IVT.(Busch et al., 1998; Janjua, Alkawi, Suri, & Qureshi, 2008; Okada, Copeland, Fitridge, Koziol, & del Zoppo, 1994) Bridging therapies with various antiplatelet agents or anticoagulants following IVT could be promising by augmenting the thrombolytic effects. (Adeoye et al., 2015; Amaro et al., 2013; Barreto et al., 2012; Jeong et al., 2016; Li et al., 2016) However, there are also concerns that such benefit could be minimized if the risk of intracranial hemorrhage is high. (Zinkstok, Roos, & ARTIS investigators, 2012).

In this study, we conducted a retrospective analysis of AIS patients who had received such combination treatment from the data in the Thrombolysis Implementation and Monitor of Acute Ischemic Stroke in China (TIMS‐China) in older to examine the efficacy and safety outcomes of intravenous heparin (IVH) immediately following IVT.

## 2 METHODS

### 2.1 Study population

TIMS‐China was a national prospective consecutive stroke registry of IVT for AIS patients in 67 major stroke centers in China. (Liao et al., 2014) None of the patients received bridging mechanical thrombectomy or other emergency endovascular treatment. The study protocol was approved by the Ethics Committee of Beijing Tiantan Hospital. The registry was regularly monitored independently by the Quality Monitoring Committee of TIMS‐China and a Contract Research Organization. For a patient with AIS eligible for enrollment, the patient or patient's legally authorized representative would be given the written informed consent of TIMS‐China study before enrollment, and all patients received IVT in the dose range between 0.5–0.9 mg/kg, with 10% of the total dose given as a bolus over 1 min and the remainder infused over 60 min. The National Institutes of Health Stroke Scale (NIHSS) score was measured at baseline, 2 hr, 24 hr, 7 days (or at discharge, whichever occurs first) and any time of neurological deterioration. The modified Rankin Scale (mRS) score was assessed at 7 days (or at discharge, whichever occurs first) and 90 days. Only the neurologists who were trained and qualified for using NIHSS and mRS recorded the scores. Brain imaging (CT or MR) was performed at baseline, 24 hr, 7 days (or at discharge, whichever occurs first) and any time of neurological deterioration. The imaging findings were interpreted by two independent trained radiologists blinded to clinical data in each participating hospital. A third experienced senior radiologist was involved to resolve any disagreement.

In the TIMS‐China registry, antithrombotic regimens after IVT were decided by an individual clinical decision. The patients who received IVH immediately after IVT (early IVH group) and those who initiated ATT until 24 hr after IVT based on the current guidelines’ recommendation (standard ATT group) were screened for this comparison. For safety reasons, patients were re‐examined for head CT after completion of 1‐hr tPA infusion and before initiation of IVH. Heparin was administered only if the patient had no intracranial or major extracranial hemorrhage. An informed consent was signed prior to initiation of IVH, and then, IVH was started as a bolus of 30 U/kg (maximum dose: 3,000 U) within a minute, followed by a continuous infusion at 500–1000 U per hour for at least 24 hr, up to 72 hr. The goal of the activated partial thromboplastin time (APTT) was 1.5–2 times of the baseline. It was monitored at baseline, 2 hr, 6 hr, 12 hr, and every 12 hr thereafter during heparin infusion and within 2 hr of any dosage adjustment. IVH was discontinued immediately when major extracranial or any intracranial bleeding was suspected.

### 2.2 Outcome measurement

The efficacy outcome measures included neurological improvement at 24 hr and 7 days, excellent recovery and functional independence at 90 days. Neurological improvement was defined as NIHSS score decrease of ≥4 points from the baseline (National Institute of Neurological Disorders and Stroke rt‐PA Stroke Study Group, 1995). The mRS was used to assess functional outcome at 90 days. A central follow‐up blinded to baseline information was carried out by telephone interview by trained neurologists based on a standardized interview protocol. Excellent recovery was defined as having a mRS score of 0–1, and functional independence was defined as having a mRS score of 0–2 (Hacke et al., 2008).

The safety outcome measures included post‐IVT symptomatic intracranial hemorrhage (sICH) and any intracranial hemorrhage (aICH) within 7 days, and mortality within 90 days. sICH was evaluated by using the European Cooperative Acute Stroke Study (ECASS) definition,(Hacke et al., 2008) which was defined as any intracranial hemorrhage associated with 4 or more points of worsening on NIHSS. aICH was verified by follow‐up imaging studies regardless of any clinical deterioration.

### 2.3 Statistical analysis

The baseline variables were compared between both groups by using a univariate analysis. The *t* test or Mann–Whitney U test was used to compare means or medians for continuous variables. The Pearson chi‐square test or Fisher's exact test was used to compare the proportions for categorical variables. In order to improve the baseline comparability between both groups in this study, we performed a propensity score (PS) matching (1:2). The probabilities of receiving early IVH were calculated by using a logistic regression model. Fourteen potential confounding covariates were used to generate PS. They were age, sex, hypertension, diabetes mellitus, atrial fibrillation, prior stroke, prestroke mRS score, cigarette smoking, systolic blood pressure, admission NIHSS score, onset‐to‐needle time, tPA dose, stroke territory, and stroke subtype by Trial of Org 10172 in Acute Stroke Treatment (TOAST) criteria. The C‐statistic was calculated to evaluate the goodness‐of‐fit for the model. After PS generation, the patients treated with early IVH and those treated with standard ATT delay of 24 hr were performed with the use of a 1:2 matching protocol without replacement (greedy‐matching algorithm), with a caliper width ≤0.2 of the standard deviation of the logit of the PS. (Bangalore et al., 2015) In the matched population, for comparing the outcomes between the two groups, the odds ratios (OR) with their 95% confidence intervals (CI) were analyzed by a binary logistic regression model. In addition, we assessed whether the effects of the two antithrombotic regimens after IVT on the mRS scores at 90 days differed in certain subgroups by testing the treatment‐by‐subgroup interaction effect with the use of an ordinal logistic regression model. Statistical significance was set at *p* < .05. All statistical analyses were performed with the statistical software package R (http://www.R‐project.org, The R Foundation) and Empowerstats (http://www.empowerstats.com, X&Y Solutions, Inc).

## 3 RESULTS

**Figure 1:**
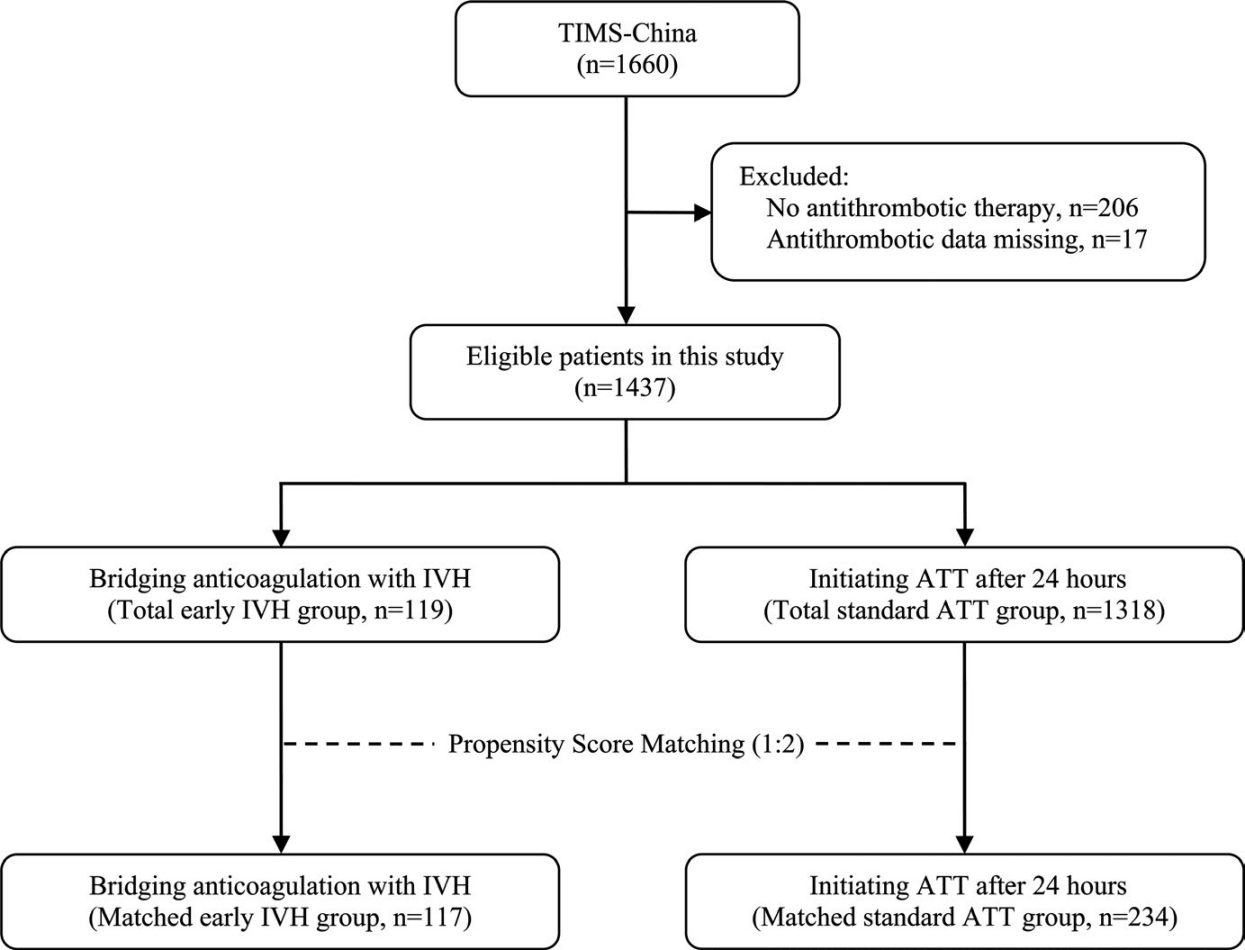
Flowchart of patient selection. Abbreviations: ATT, antithrombotic therapy, IVH, intravenous heparin, TIMS‐China, Thrombolysis Implementation and Monitor of Acute Ischemic Stroke in China

From May 2007 to February 2015, TIMS‐China database consecutively registered 1660 AIS patients treated with IVT. A total of 206 patients who had not been given any antithrombotic drugs after IVT and 17 patients with data missing on antithrombotic therapy were excluded. Finally, 1,437 patients were eligible to be entered into the current study. The early IVH group had 119 (8.3%) cases, and the standard ATT group had 1,318 (91.7%) cases. After PS matching (1:2), 117 cases in the early IVH group matched 234 cases in the standard ATT group and were selected for analysis (Figure 1). At 90 days, a total of 4 patients (1.1%) were lost to follow‐up in the matched population.

Tables 1, 2 showed the baseline characteristics of the heparin and standard ATT groups in the total and matched study population, respectively. In the total population, the baseline characteristics were markedly imbalanced between the heparin and standard ATT groups. The patients in the early IVH group had higher baseline systolic blood pressure (mean: 155 vs. 148 mmHg, *p* < .01), more severe strokes (median NIHSS scores at admission: 14 vs. 10, *p* < .01), and delayed use of IVT (median onset‐to‐needle time: 4 vs. 3 hr, *p* < .01) compared to those in the standard ATT group. Furthermore, the IVH‐treated patients had more often past history of stroke (26.9% vs. 17.4%, *p* = .01), less use of full dose tPA (58.8% vs. 71.1%, *p* = .01), and anterior circulation strokes (56.3% vs. 84.7%, *p* < .01) than those given standard ATT. After PS matching, the baseline characteristics of 117‐pair (1:2) patients between the two groups were homogeneous (Table 2). The C‐statistic of goodness‐of‐fit for PS model was 0.74 (95% CI = 0.69–0.79), which represented the area under the receiver operating characteristic curve and indicated moderate discrimination between both groups.

**Table 1:** Baseline characteristics of heparin versus standard ATT group in the total population

Baseline variable	Early IVH group (*n* = 119)	Standard ATT group (*n* = 1,318)	P value
Age, mean (*SD*), year	60 (9)	62 (12)	0.06
Male sex	84 (70.6)	841 (63.8)	0.14
Hypertension	73 (61.3)	762 (57.8)	0.46
Diabetes mellitus	18 (15.1)	232 (17.6)	0.50
Atrial fibrillation	15 (12.6)	200 (15.2)	0.45
Prior stroke	32 (26.9)	229 (17.4)	0.01
Prestroke mRS score > 1	4 (3.4)	44 (3.3)	> 0.99
Cigarette smoking	49 (41.2)	575 (43.6)	0.61
Systolic blood pressure, mean (*SD*), mmHg	155 (23)	148 (20)	< 0.01
Admission NIHSS score, median (IQR)	14 (10–19)	10 (7–15)	< 0.01
Onset‐to‐needle time, median (IQR), hour	4.0 (3.0–4.9)	3.0 (2.5–4.3)	< 0.01
Full dose of tPA	70 (58.8)	937 (71.1)	0.01
Stroke territory^a^			< 0.01
Anterior circulation	67 (56.3)	966 (84.7)	
Posterior circulation	52 (43.7)	174 (15.3)	
Stroke subtype by TOAST criteria^b^			0.21
Large artery atherosclerosis	69 (58.0)	759 (57.8)	
Small artery occlusion	19 (16.0)	135 (10.3)	
Cardioembolism	16 (13.4)	214 (16.3)	
Other determined or undetermined etiology	15 (12.6)	206 (15.7)	

Note

Values are numbers with percentages in parentheses, unless indicated otherwise.

Abbreviations: ATT, antithrombotic therapy, IQR, interquartile range, IVH, intravenous heparin, mRS, modified Rankin Scale, NIHSS, National Institutes of Health Stroke Scale, *SD*, standard deviation, TOAST, Trial of Org 10,172 in Acute Stroke Treatment, tPA, tissue Plasminogen Activator.

^a^ Thirteen cases with stroke in both anterior and posterior circulation territories and 165 cases with unclear stroke territory in the standard ATT group.

^b^ Four missing values in the standard ATT group.

**Table 2:** Baseline characteristics of heparin versus. standard ATT group in the matched population

Baseline variable	Early IVH group (*n* = 117)	Standard ATT group (*n* = 234)	P value
Age, mean (*SD*), year	61 (9)	60 (12)	0.81
Male sex	83 (70.3)	171 (73.1)	0.67
Hypertension	71 (60.7)	142 (60.7)	1.00
Diabetes mellitus	18 (15.4)	36 (15.4)	1.00
Atrial fibrillation	14 (12.0)	27 (11.5)	0.91
Prior stroke	32 (27.4)	64 (27.4)	1.00
Prestroke mRS score > 1	4 (3.4)	11 (4.7)	0.78
Cigarette smoking	48 (41.0)	99 (42.3)	0.82
Systolic blood pressure, mean (*SD*), mmHg	155 (23)	155 (21)	0.91
Admission NIHSS score, median (IQR)	14 (10–18)	14 (8–20)	0.49
Onset‐to‐needle time, median (IQR), hour	4.0 (3.0–4.8)	4.0 (2.5–4.9)	0.85
Full dose of tPA	69 (59.0)	142 (60.7)	0.76
Stroke territory			1.00
Anterior circulation	67 (57.3)	134 (57.3)	
Posterior circulation	50 (42.7)	100 (42.7)	
Stroke subtype by TOAST criteria			0.57
Large artery atherosclerosis	68 (58.1)	154 (65.8)	
Small artery occlusion	19 (16.2)	30 (12.8)	
Cardioembolism	15 (12.8)	25 (10.7)	
Other determined or undetermined etiology	15 (12.8)	25 (10.7)	

Note

Values are numbers with percentages in parentheses, unless indicated otherwise.

Abbreviations: ATT, antithrombotic therapy, IQR, interquartile range, IVH, intravenous heparin, mRS, modified Rankin Scale, NIHSS, National Institutes of Health Stroke Scale, *SD*, standard deviation, TOAST, Trial of Org 10,172 in Acute Stroke Treatment, tPA, tissue Plasminogen Activator.

**Figure 2:**
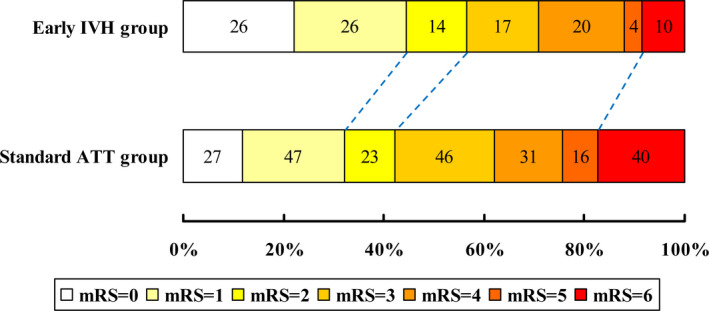
The mRS distribution of early IVH versus standard ATT group in the matched population. Abbreviations: ATT, antithrombotic therapy, IVH, intravenous heparin, mRS, modified Rankin Scale

Table 3 showed the efficacy and safety outcomes of the heparin and standard ATT groups in the matched population. The binary logistic regression analysis demonstrated that the proportions of neurological improvement at 24 hr (53.0% vs. 33.5%, OR = 2.24, 95% CI = 1.42–3.53, *p* < .01) and 7 days (63.2% vs. 47.2%, OR = 1.92, 95% CI = 1.22–3.03, *p* < .01) were significantly higher in the early IVH group than in the standard ATT group. Moreover, patients of early IVH group had not only lower incidences of 90‐day mortality (8.5% vs. 17.4%, OR = 0.44, 95% CI = 0.21–0.92, *p* = .03) but also better chances of excellent recovery (44.4% vs. 32.2%, OR = 1.69, 95% CI = 1.07–2.67, *p* = .03) and functional independence (56.4% vs. 42.2%, OR = 1.77, 95% CI = 1.13–2.78, *p* = .01) at 90 days than those of standard ATT group. The distribution of 90‐day mRS scores between the heparin and control groups was shown in Figure 2. However, IVH combined with IVT did not seem to significantly increase the risk of sICH (5.1% vs. 5.6%, OR = 0.92, 95% CI = 0.34–2.48, *p* = .87) and aICH (22.2% versus. 15.4%, OR = 1.57, 95% CI = 0.90–2.76, *p* = .12).

**Table 3:** Outcomes of heparin versus. standard ATT group in the matched population

Outcome variable	Early IVH group	Standard ATT group	Binary logistic analysis	
			OR (95% CI)	P value
**Efficacy outcome**				
Neurological improvement at 24 hr	62/117 (53.0)	78/233 (33.5)	2.24 (1.42, 3.53)	<0.01
Neurological improvement at 7 d^a^	74/117 (63.2)	110/233 (47.2)	1.92 (1.22, 3.03)	<0.01
Excellent recovery at 90 d	52/117 (44.4)	74/230 (32.2)	1.69 (1.07, 2.67)	0.03
Functional independence at 90 d	66/117 (56.4)	97/230 (42.2)	1.77 (1.13, 2.78)	0.01
**Safety outcome**				
sICH within 7 d^a^	6/117 (5.1)	13/234 (5.6)	0.92 (0.34, 2.48)	0.87
aICH within 7 d^a^	26/117 (22.2)	36/234 (15.4)	1.57 (0.90, 2.76)	0.12
Mortality within 90 d	10/117 (8.5)	40/230 (17.4)	0.44 (0.21, 0.92)	0.03

Note

Data are event number/total number (%), unless otherwise indicated.

sICH was evaluated by using the European Cooperative Acute Stroke Study (ECASS) definition, which was defined as any intracranial hemorrhage associated with 4 or more points of worsening on NIHSS. aICH was verified by follow‐up imaging studies regardless of any clinical deterioration. Neurological improvement was defined as NIHSS score decrease of ≥ 4 points from the baseline. Excellent recovery was defined as having a mRS score of 0–1, and functional independence was defined as having a mRS score of 0–2.

Abbreviations: aICH, any intracranial hemorrhage, ATT, antithrombotic therapy, CI, confidence interval, IVH, intravenous heparin, OR, odds ratio, sICH, symptomatic intracranial hemorrhage.

^a^ At 7 d or discharge, whichever comes first.

Table 4 showed stratified analyses of 90‐day mRS between heparin and standard ATT groups in the matched population. The ordinal logistic regression analysis demonstrated that there was no significant interaction effect on 90‐day mRS score between different post‐IVT antithrombotic regimens and all subgroups stratified by age, sex, admission NIHSS score, onset‐to‐needle time, tPA dose, stroke territory, and stroke subtype (*p* > .10 for all interactions).

**Table 4:** Stratified analyses of 90‐day mRS between heparin and standard ATT groups in the matched population

Subgroup	No. of patients	Early IVH group	Standard ATT group	Common OR^a^ (95% CI)	P for interaction
		mRS at 90 days, median (IQR)			
Age					0.70
≤ 60 years	171	2 (1–3)	3 (1–4)	1.82 (1.01–3.25)	
> 60 years	180	2 (1–4)	3 (1–5)	2.16 (1.15–4.06)	
Sex					0.31
Male	254	2 (1–4)	3 (1–4)	1.72 (1.03–2.86)	
Female	97	3 (0–4)	4 (1–5)	2.80 (1.21–6.49)	
Atrial fibrillation					0.49
Yes	41	2 (1–3)	4 (1–6)	2.97 (0.79–11.36)	
No	310	2 (1–4)	3 (1–4)	1.86 (1.16–2.94)	
Admission NIHSS score					0.12
< 15 points	185	1 (0–3)	2 (1–3)	1.42 (0.87–2.32)	
≥ 15 points	166	3 (1–4)	4 (3–6)	2.64 (1.40–5.00)	
Onset‐to‐needle time					0.25
≤ 3 hr	142	2 (0–4)	3 (1–5)	2.75 (1.20–6.30)	
> 3 hr	209	2 (1–4)	3 (1–4)	1.57 (0.93–2.64)	
tPA dose					0.28
Full dose (0.9mg/kg, max = 90mg)	211	2 (1–4)	3 (1–4)	1.62 (0.90–2.86)	
Low dose (0.5–0.8mg/kg, max = 50mg)	140	2 (0–3)	3 (1–5)	2.64 (1.36–5.10)	
Stroke territory					0.65
Anterior circulation	201	2 (1–3)	3 (1–4)	1.79 (1.08–2.97)	
Posterior circulation	150	2 (0–4)	3 (1–6)	2.18 (1.02–4.66)	
Stroke subtype					0.66
Large artery atherosclerosis	222	2 (1–4)	3 (1–5)	1.99 (1.14–3.49)	
Non‐large artery atherosclerosis	129	2 (0–3)	2 (1–4)	1.65 (0.84–3.22)	

Abbreviations: ATT, antithrombotic therapy, CI, confidence interval, IQR, interquartile range, IVH, intravenous heparin, mRS, modified Rankin Scale, NIHSS, National Institutes of Health Stroke Scale, TOAST, Trial of Org 10,172 in Acute Stroke Treatment, tPA, tissue Plasminogen Activator.

^a^ The common OR values were calculated using an ordinal logistic regression model and indicated the odds of improvement of 1 point on the mRS at 90 days.

## 4 DISCUSSION

This matched case–control study has demonstrated that AIS patients who received early IVH following IVT not only had a significant improvement of the NIHSS score at 24 hr and 7 days from baseline, but also achieved a better 90‐day functional outcome, without increasing their risk of intracranial hemorrhage. On the issue of IVT combined with IVH for AIS, as early as 1992, a small pilot study was conducted to explore the safety of intravenous tPA and heparin in acute middle cerebral artery stroke. It was found that the incidence of deleterious hemorrhage was <10%, within acceptable safety limits. (von Kummer & Hacke, 1992) From 1998 to 2013, two single‐arm cohorts compared with historical controls and a single‐center case–control study demonstrated that thrombolysis followed by anticoagulation with IVH did not increase the risk of sICH. (Amaro et al., 2013; Grond et al., 1998; Schmülling et al., 2003) As for patients with acute basilar artery stroke, one study showed a higher rate of sICH and a shift toward worse outcome in thrombolyzed patients treated with IVH as compared to subcutaneous low‐molecular weight heparin,(Ritvonen et al., 2019) while another study did not find that sICH after thrombolysis was related to adjuvant anticoagulation with IVH. (Sairanen et al., 2015) These observational case series showed conflicting results largely because of inhomogeneous selection of patients and different dosage of heparin for the study.

TIMS‐China was a real‐world study on intravenous thrombolysis with tPA for AIS in China, the study collected the dosage of tPA from routine clinical settings. Influenced by Japanese studies,(Nakagawara et al., 2010) many hospitals in Asia, including China, have used low‐dose IVT to treat AIS patients. In addition, some Chinese neurologists prefer to use low‐dose tPA (maximum: 50mg) in order to reduce the risk of bleeding and save money for patients. In our study of 351 patients in the matched population, 140 cases (39.9%) received lower doses of tPA (0.5–0.8 mg/kg, maximum: 50 mg). Although previous studies from Japan found that the efficacy and safety of the low‐dose tPA (0.6 mg/kg) was comparable to that of the full dose (0.9 mg/kg), (Minematsu et al., 2012) lower doses of tPA could weaken the potency of tPA (Anderson et al., 2016; Liao et al., 2014). By using an ordinal logistic analysis, the interaction of IVH with different doses of IVT was studied. Our analysis showed that there was no significant interaction effect on 90‐day mRS between heparin and standard ATT groups when stratified by different tPA doses.

Our study had several limitations. Firstly, this study was a retrospective analysis of prospectively collected data and was thus vulnerable to selection bias, despite the fact that important baselines in both groups were well‐balanced by using a PS matching. However, the C‐statistic for PS model was as low as 0.74, some unmeasured factors might affect physician's decision on the selection of early IVH. For example, clinical experience and research suggest that several potential factors could increase the risk of hemorrhagic transformation after thrombolysis include large infarcts, early ischemic changes, poor collaterals, multiple microbleeds, high glucose, renal impairment, and previous antiplatelet agents (Charidimou et al., 2017; Whiteley, Slot, Fernandes, Sandercock, & Wardlaw, 2012). All of these factors can be recognized by treating physicians before the initiation of IVH. By balancing the risks and benefits, IVH was likely to be administered to a certain subset of patients who might benefit more. In our analyzed cases, early heparin was often used in patients having received low‐dose tPA, with more severe strokes or posterior circulation strokes. It is also possible that early use of IVH was decided by the unknown intermediate factors, presumably improving functional outcomes and decreasing bleeding risks. Moreover, the rate of intracranial hemorrhage in this analysis may be underestimated because the patients were excluded due to ultra‐early hemorrhage after thrombolysis that withholds initial use of antithrombotics. Secondly, there was no follow‐up imaging assessment in the database to support the notion of possible vessel recanalization. Last but not least, our results were obtained in a subgroup of Chinese AIS patients with moderate severity strokes (median NIHSS = 14), with low‐dose tPA used in 40% and posterior circulation strokes in 43% of cases, and cannot be extrapolated to other population groups.

## 5 CONCLUSIONS

In summary, this matched‐control study aimed to investigate the effect of early IVH after IVT on the clinical outcomes in a certain subset of AIS patients, which is not recommended by the current guidelines. In this retrospective analysis from a prospective registry, such combination therapy with IVT followed by IVH might improve their functional outcomes and not increase their risk of hemorrhagic transformation. Nevertheless, future randomized placebo‐controlled multicenter trials are needed to confirm our findings.

## CONFLICTS OF INTEREST

All authors have no conflicts of interest to disclose.

## **AUTHOR** **CONTRIBUTION**

Xu Tong and Yilong Wang involved in study concept and design; Xu Tong and Yibin Cao involved in acquisition of data; Wenzhi Wang involved in analysis of data; Yilong Wang involved in interpretation of data; Yongjun Wang and Yilong Wang involved in study supervision; David Wang and Yilong Wang involved in critical revision of manuscript for intellectual content.

### Peer Review

The peer review history for this article is available at https://publons.com/publon/10.1002/brb3.1776.

## Data Availability

The raw data supporting the conclusions of this manuscript will be made available by the author Yilong Wang (yilong528@gmail.com), without undue reservation, to any qualified researcher.

## References

[brb31776-bib-0001] Adeoye, O. , Sucharew, H. , Khoury, J. , Vagal, A. , Schmit, P. A. , Ewing, I. , … Pancioli, A. M. (2015). Combined approach to lysis utilizing eptifibatide and recombinant tissue‐type plasminogen activator in acute ischemic stroke‐full dose regimen stroke trial. Stroke, 46, 2529–2533. 10.1161/STROKEAHA.115.010260 26243231 PMC4550507

[brb31776-bib-0002] Alexandrov, A. V. , & Grotta, J. C. (2002). Arterial reocclusion in stroke patients treated with intravenous tissue plasminogen activator. Neurology, 59, 862–867. 10.1212/WNL.59.6.862 12297567

[brb31776-bib-0003] Amaro, S. , Llull, L. , Urra, X. , Obach, V. , Cervera, Á. , & Chamorro, Á. (2013). Risks and benefits of early antithrombotic therapy after thrombolytic treatment in patients with acute stroke. PLoS One, 8, e71132 10.1371/journal.pone.0071132 23951093 PMC3738638

[brb31776-bib-0004] Anderson, C. S. , Robinson, T. , Lindley, R. I. , Arima, H. , Lavados, P. M. , Lee, T. H. , … ENCHANTED Investigators and Coordinators . (2016). ENCHANTED Investigators and Coordinators. Low‐Dose versus Standard‐Dose Intravenous Alteplase in Acute Ischemic Stroke. New England Journal of Medicine, 374, 2313–2323. 10.1056/NEJMoa1515510 27161018

[brb31776-bib-0005] Bangalore, S. , Guo, Y. , Samadashvili, Z. , Blecker, S. , Xu, J. , & Hannan, E. L. (2015). Everolimus‐eluting stents or bypass surgery for multivessel coronary disease. New England Journal of Medicine, 372, 1213–1222. 10.1056/NEJMoa1412168 25775087

[brb31776-bib-0006] Barreto, A. D. , Alexandrov, A. V. , Lyden, P. , Lee, J. , Martin‐Schild, S. , Shen, L. , … Grotta, J. C. (2012). The argatroban and tissue‐type plasminogen activator stroke study: Final results of a pilot safety study. Stroke, 43, 770–775. 10.1161/STROKEAHA.111.625574 22223235 PMC3289043

[brb31776-bib-0007] Busch, E. , Krüger, K. , Allegrini, P. R. , Kerskens, C. M. , Gyngell, M. L. , Hoehn‐Berlage, M. , & Hossmann, K. A. (1998). Reperfusion after thrombolytic therapy of embolic stroke in the rat: Magnetic resonance and biochemical imaging. Journal of Cerebral Blood Flow and Metabolism, 18, 407–418. 10.1097/00004647-199804000-00009 9538906

[brb31776-bib-0008] Charidimou, A. , Turc, G. , Oppenheim, C. , Yan, S. , Scheitz, J. F. , Erdur, H. , … Werring, D. J. , 2017). Microbleeds, cerebral hemorrhage, and functional outcome after stroke thrombolysis. Stroke, 48, 2084–2090. 10.1161/STROKEAHA.116.012992 28720659

[brb31776-bib-0009] Fassbender, K. , Dempfle, C. E. , Mielke, O. , Schwartz, A. , Daffertshofer, M. , Eschenfelder, C. , … Hennerici, M. (1999). Changes in coagulation and fibrinolysis markers in acute ischemic stroke treated with recombinant tissue plasminogen activator. Stroke, 30, 2101–2104. 10.1161/01.STR.30.10.2101 10512913

[brb31776-bib-0010] Grond, M. , Stenzel, C. , Schmülling, S. , Rudolf, J. , Neveling, M. , Lechleuthner, A. , … Heiss, W.‐D. (1998). Early intravenous thrombolysis for acute ischemic stroke in a community‐based approach. Stroke, 29(8), 1544–1549. 10.1161/01.STR.29.8.1544 9707190

[brb31776-bib-0011] Hacke, W. , Kaste, M. , Bluhmki, E. , Brozman, M. , Dávalos, A. , Guidetti, D. , … Toni, D. (2008). Thrombolysis with alteplase 3 to 4.5 hours after acute ischemic stroke. New England Journal of Medicine, 359(13), 1317–1329. 10.1056/NEJMoa0804656 18815396

[brb31776-bib-0012] Janjua, N. , Alkawi, A. , Suri, M. F. , & Qureshi, A. I. (2008). Impact of arterial reocclusion and distal fragmentation during thrombolysis among patients with acute ischemic stroke. AJNR. American Journal of Neuroradiology, 29, 253–258. 10.3174/ajnr.A0825 18024576 PMC8119013

[brb31776-bib-0013] Jeong, H. G. , Kim, B. J. , Yang, M. H. , Han, M. K. , Bae, H. J. , & Lee, S. H. (2016). Stroke outcomes with use of antithrombotics within 24 hours after recanalization treatment. Neurology, 87, 996–1002. 10.1212/WNL.0000000000003083 27521435

[brb31776-bib-0014] Li, W. , Lin, L. , Zhang, M. , Wu, Y. , Liu, C. , Li, X. , … Feng, W. (2016). Safety and preliminary efficacy of early tirofiban treatment after alteplase in acute ischemic stroke patients. Stroke, 47, 2649–2651. 10.1161/STROKEAHA.116.014413 27608821

[brb31776-bib-0015] Liao, X. , Wang, Y. , Pan, Y. , Wang, C. , Zhao, X. , Wang, D. Z. , … Wang, Y. (2014). Standard‐dose intravenous tissue‐type plasminogen activator for stroke is better than low doses. Stroke, 45(8), 2354–2358. 10.1161/STROKEAHA.114.005989 25013020

[brb31776-bib-0016] Minematsu, K. , Toyoda, K. , Hirano, T. , Kimura, K. , Kondo, R. , Mori, E. , … Japan Stroke Society (2012). Guidelines for the intravenous application of recombinant tissue‐type plasminogen activator (alteplase), the second edition, October 2012: a guideline from the Japan Stroke Society. Journal of Stroke and Cerebrovascular Diseases, 2013(22), 571–600. 10.1016/j.jstrokecerebrovasdis.2013.04.001 23727456

[brb31776-bib-0017] Muchada, M. , Rodriguez‐Luna, D. , Pagola, J. , Flores, A. , Sanjuan, E. , Meler, P. , … Rubiera, M. (2014). Impact of time to treatment on tissue‐type plasminogen activator‐induced recanalization in acute ischemic stroke. Stroke, 45, 2734–2738. 10.1161/STROKEAHA.114.006222 25104845

[brb31776-bib-0018] Nakagawara, J. , Minematsu, K. , Okada, Y. , Tanahashi, N. , Nagahiro, S. , Mori, E. , … Investigators, J.‐M.‐A.‐R.‐S. (2010). Thrombolysis with 0.6 mg/kg intravenous alteplase for acute ischemic stroke in routine clinical practice: The Japan post‐Marketing Alteplase Registration Study (J‐MARS). Stroke, 41, 1984–1989. 10.1161/STROKEAHA.110.589606 20651262

[brb31776-bib-0019] National Institute of Neurological Disorders and Stroke rt‐PA Stroke Study Group (1995). Tissue plasminogen activator for acute ischemic stroke. New England Journal of Medicine 333(24), 1581–1587. 7477192 10.1056/NEJM199512143332401

[brb31776-bib-0020] Okada, Y. , Copeland, B. R. , Fitridge, R. , Koziol, J. A. , & del Zoppo, G. J. (1994). Fibrin contributes to microvascular obstructions and parenchymal changes during early focal cerebral ischemia and reperfusion. Stroke, 25(9), 1847–1853. discussion 1853–4. 10.1161/01.STR.25.9.1847 8073468

[brb31776-bib-0021] Powers, W. J. , Rabinstein, A. A. , Ackerson, T. et al (2018). 2018 guidelines for the early management of patients with acute ischemic stroke: a guideline for healthcare professionals from the American Heart Association/American Stroke Association. Stroke, 49(3), e46–e110. 29367334 10.1161/STR.0000000000000158

[brb31776-bib-0022] Rha, J. H. , & Saver, J. L. (2007). The impact of recanalization on ischemic stroke outcome: A meta‐analysis. Stroke, 38, 967–973. 10.1161/01.STR.0000258112.14918.24 17272772

[brb31776-bib-0023] Ritvonen, J. , Strbian, D. , Silvennoinen, H. , Virtanen, P. , Salonen, O. , Lindsberg, P. J. , & Sairanen, T. (2019). Thrombolysis and adjunct anticoagulation in patients with acute basilar artery occlusion. European Journal of Neurology, 26, 128–135. 10.1111/ene.13781 30134080

[brb31776-bib-0024] Rubiera, M. , Alvarez‐Sabín, J. , Ribo, M. , Montaner, J. , Santamarina, E. , Arenillas, J. F. , … Molina, C. A. (2005). Predictors of early arterial reocclusion after tissue plasminogen activator‐induced recanalization in acute ischemic stroke. Stroke, 36, 1452–1456. 10.1161/01.STR.0000170711.43405.81 15947260

[brb31776-bib-0025] Sairanen, T. , Strbian, D. , Ruuskanen, R. , Silvennoinen, H. , Salonen, O. , & Lindsberg, P. J. (2015). Symptomatic intracranial haemorrhage after thrombolysis with adjuvant anticoagulation in basilar artery occlusion. European Journal of Neurology, 22, 493–499. 10.1111/ene.12597 25482105

[brb31776-bib-0026] Schmülling, S. , Rudolf, J. , Strotmann‐Tack, T. , Grond, M. , Schneweis, S. , Sobesky, J. , … Heiss, W.‐D. , 2003). Acetylsalicylic acid pretreatment, concomitant heparin therapy and the risk of early intracranial hemorrhage following systemic thrombolysis for acute ischemic stroke. Cerebrovascular Diseases, 16, 183–190. 10.1159/000071114 12865603

[brb31776-bib-0027] von Kummer, R. , & Hacke, W. (1992). Safety and efficacy of intravenous tissue plasminogen activator and heparin in acute middle cerebral artery stroke. Stroke, 23, 646–652. 10.1161/01.STR.23.5.646 1579960

[brb31776-bib-0028] Whiteley, W. N. , Slot, K. B. , Fernandes, P. , Sandercock, P. , & Wardlaw, J. (2012). Risk factors for intracranial hemorrhage in acute ischemic stroke patients treated with recombinant tissue plasminogen activator: A systematic review and meta‐analysis of 55 studies. Stroke, 43, 2904–2909. 10.1161/STROKEAHA.112.665331 22996959

[brb31776-bib-0029] Yeo, L. L. L. , Paliwal, P. , Teoh, H. L. , Seet, R. C. , Chan, B. P. L. , Liang, S. , … Sharma, V. K. , 2013). Timing of recanalization after intravenous thrombolysis and functional outcomes after acute ischemic stroke. JAMA Neurology, 70, 353–358. 10.1001/2013.jamaneurol.547 23599933

[brb31776-bib-0030] Zinkstok, S. M. , Roos, Y. B. , & ARTIS investigators . (2012). Early administration of aspirin in patients treated with alteplase for acute ischaemic stroke: A randomised controlled trial. Lancet, 380, 731–737. 10.1016/S0140-6736(12)60949-0 22748820

